# Breast cancer patients enrolled in the Swiss mammography screening program “donna” demonstrate prolonged survival

**DOI:** 10.1186/s13058-024-01841-6

**Published:** 2024-05-27

**Authors:** David Kuklinski, Marcel Blum, Jonas Subelack, Alexander Geissler, Alena Eichenberger, Rudolf Morant

**Affiliations:** 1 Chair of Health Economics, Policy and Management, School of Medicine, University of St. Gallen, St. Jakobstr. 21, 9000 St. Gallen, Switzerland; 2 Cancer League of Eastern Switzerland, St. Gallen, Switzerland

**Keywords:** Mammography screening program, Breast cancer, Survival, Mastectomy, Switzerland

## Abstract

**Study goal:**

We compared the survival rates of women with breast cancer (BC) detected within versus outside the mammography screening program (MSP) “donna”.

**Methods:**

We merged data from the MSP with the data from corresponding cancer registries to categorize BC cases as within MSP (screen-detected and interval carcinomas) and outside the MSP. We analyzed the tumor stage distribution, tumor characteristics and the survival of the women. We further estimated hazard ratios using Cox-regressions to account for different characteristics between groups and corrected the survival rates for lead-time bias.

**Results:**

We identified 1057 invasive (ICD-10: C50) and in-situ (D05) BC cases within the MSP and 1501 outside the MSP between 2010 and 2019 in the Swiss cantons of St. Gallen and Grisons. BC within the MSP had a higher share of stage I carcinoma (46.5% vs. 33.0%; *p* < 0.01), a smaller (mean) tumor size (19.1 mm vs. 24.9 mm, *p* < 0.01), and fewer recurrences and metastases in the follow-up period (6.7% vs. 15.6%, *p* < 0.01). The 10-year survival rates were 91.4% for women within and 72.1% for women outside the MSP (*p* < 0.05). Survival difference persisted but decreased when women within the same tumor stage were compared. Lead-time corrected hazard ratios for the MSP accounted for age, tumor size and Ki-67 proliferation index were 0.550 (95% CI 0.389, 0.778; *p* < 0.01) for overall survival and 0.469 (95% CI 0.294, 0.749; *p* < 0.01) for BC related survival.

**Conclusion:**

Women participating in the “donna” MSP had a significantly higher overall and BC related survival rate than women outside the program. Detection of BC at an earlier tumor stage only partially explains the observed differences.

## Introduction

The goal of a mammography screening program (MSP) is to detect breast cancer (BC) at a prognostically favorable early stage, thus reducing the burden of disease by less aggressive treatment as well as increasing survival rates [1]. Randomized controlled trials (RCT) from the 1970’s and 1980’s demonstrated a reduction of BC related mortality rates by 15–20% through MSPs [2]. Consequently, national screening programs were introduced in an increasing number of countries. However, following a review by Gøtzsche et al. [3, 4] which concluded that MSPs were not cost-effective, in 2013, the Swiss Medical Board [5] officially advised against the introduction of new MSPs and recommended fading out existing ones. This recommendation was controversially discussed due to contradicting evidence from other studies [6].

While the effectiveness of MSPs is often evaluated based on studies from the past, the underlying decades-old data are only partially applicable to today’s situation. Mortality of BC has about halved in Switzerland [7], the USA [8], and many other Western countries [9]. More advanced treatment options, better technology for digital mammographies, the use of magnetic resonance imaging (MRI), vacuum assisted biopsies and wider use of screening programs as well as a higher awareness of women and the availability of specialized breast centers have all contributed to this favorable change.

To account for these improvements in diagnosis and treatment, updated studies are required using different methodologies, as conducting RCTs for the evaluation of MSPs is ethically not acceptable. Employing various methodological designs, a series of more recent international studies has shown decreased mortality and a reduction in disability-adjusted life years for women with BC diagnosed via MSPs [10–12]. For Switzerland, Herrmann [7] found a strong decline of overall BC mortality from 1992 to 2012, but, by comparing cantons with and without MSP, he did not detect significant regional differences as an effect of organized MSPs. Reports of several Swiss MSPs do not comment on mortality reduction due to screening. To the best of our knowledge a scientific evaluation on patient level of the effect on survival rates of MSPs compared to opportunistic screening was not performed in Switzerland, yet.

Therefore, we merged data from the MSP “donna” with a comprehensive dataset from the Cancer Registry of Eastern Switzerland and Grisons-Glarus to evaluate the impact of the MSP “donna” on the tumor stage distribution of detected BC, on the survival of the affected women and the applied cancer treatment. While other studies focused on effect differences between screen-detected versus non-screen-detected BC cases [13, 14], we deliberately investigated the effect that participation in an MSP had on women diagnosed with BC.

## Patients and methods

### Study population

The study included all 50- to 69-year-old women diagnosed with invasive and in-situ BC (ICD-10: C50 and D05) between 2010 and 2019 in the Swiss cantons of St. Gallen and Grisons. BC was either diagnosed within or outside the “donna” MSP. Cancer data was retrieved from the databases of the Cancer Registries of Eastern Switzerland and Grisons-Glarus [15], which document all cancer cases diagnosed in the two cantons and follow-up the vital status of each patient via the cantonal administrations and record the date of death or the date of last contact.

### The MSP “donna” in the Swiss cantons of St. Gallen and Grisons

Each woman between 50 and 69 years of age in the cantons of St. Gallen (since 2010) and Grisons (since 2011) is invited bi-annually to voluntarily participate in the MSP “donna”, free of charge except for a small deductible. Each year around 18,500 mammographies are done within the MSP, with a participation rate oscillating around 50%.

### Sample generation and data cleansing

**Fig. 1:**
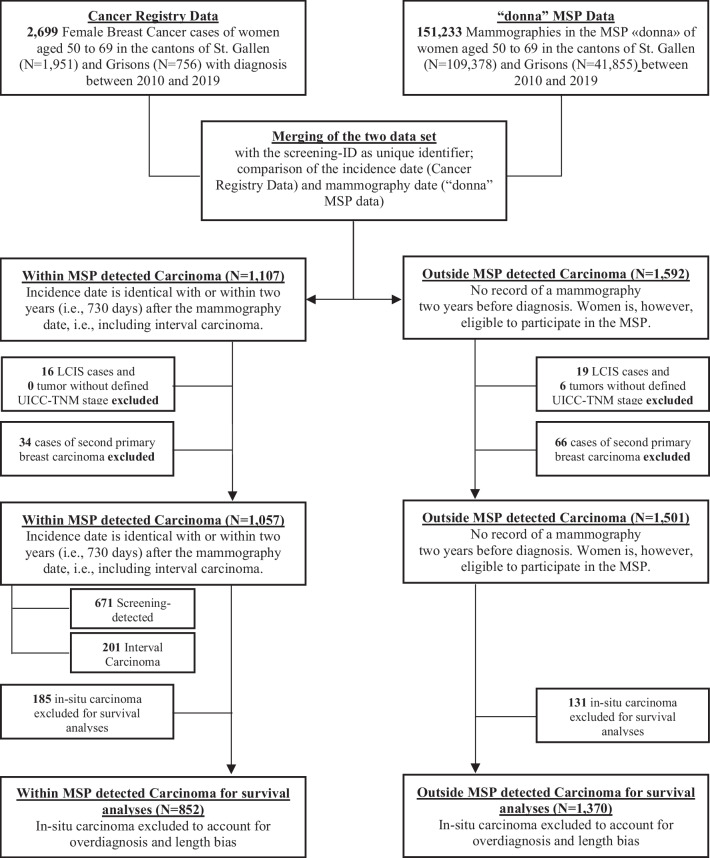
Derivation of the final data set for the analyses. Notes: Second primary BC were either excluded if diagnosed after the first primary BC or if two BCs had the same diagnosis date, the one with the more favorable tumor characteristics, based on UICC-TNM stage, T-Stage, histologic grading, tumor size, estrogen or progesterone receptor status, was excluded

Figure 1 shows the derivation of the final data sample for our analyses. The data from the Cancer Registries and from the MSP were merged using a unique screening-ID. A recorded tumor from the Cancer Registries was classified as a BC within MSP if the woman took part in the MSP “donna” and the carcinoma was either screen-detected or symptomatically detected within 2 years after the last mammography (see definition for interval carcinoma [16]). The remaining women with BC were labelled as outside the MSP. A total of 35 lobular carcinoma in situ (LCIS) cases were excluded from the analyses. Further, six cases were excluded due to insufficient information regarding their UICC-TNM tumor stage. 100 women had two primary BCs within the study period. Only the first tumor was included in the sample. If two primary tumors were diagnosed on the same day, the tumor with the highest stage was considered. Hence, the final data set comprised 1057 BC cases within and 1501 BC cases outside the MSP. The carcinomas within the MSP were either screen-detected carcinomas following a positive mammography (n = 840) or interval cancer cases (n = 217). Furthermore, we excluded in-situ carcinomas (n = 316) from the survival analyses to partially account for length bias and overdiagnosis from screening [17].

### Variables of interest

The final sample includes variables on patient and tumor characteristics, treatments, and survival. Patient characteristics include the age at diagnosis, canton of living and birthplace. Tumor characteristics include information about the biology of the tumor, i.e., tumor stage, tumor size, hormone receptor status, histologic grading, the human epidermal growth factor receptor 2 (HER2) status, and the Ki-67 proliferation index (Ki-67). Data for the patient’s primary treatments include information about adjuvant chemotherapies, surgical interventions, radiation therapy and whether the treatment was done in a certified breast center. Finally, follow-up data includes information about any occurred metastasis or recurrence, and the date of the last updated vital status. Patients from the canton of St. Gallen were followed up until mid-November 2022, from the canton of Grisons until the end of August 2023.

### Methods and statistical analyses

Carcinomas within the MSP were compared to those outside the MSP with respect to their patient and tumor characteristics, primary treatments, and their survival. Wilcoxon-Mann Whitney rank sum tests or student’s t-tests, as appropriate, were used to test for significance in differences. The survival of the two groups was compared using Kaplan–Meier curves. Greenwood Point estimators for the Kaplan–Meier estimates were used to test for significance of the results.

Furthermore, to account for confounders, Cox proportional hazards regression models were calculated to estimate hazard ratios (HR). We estimated models controlling for age at diagnosis, tumor stages, Ki-67, tumor size, HER2, and treatment in certified breast centers to investigate potential survival differences. Moreover, we corrected results from Kaplan–Meier estimates and Cox proportional hazards regressions for lead-time bias, by adjusting survival time of women whose BC was screen-detected. Corrections are based on the methodological approach of Duffy et al. [13], using mean sojourn times from Tabar et al. [18]. We further included the year of diagnosis as dummy to account for treatment advancement over time (incidence period correction). All statistical analyses were done in Stata 18.

### Ethics approval

This retrospective cohort study was submitted to the Ethics Commission of Eastern Switzerland (EKOS-23/061). It was ruled to be a quality control covered by the informed patient consent of the MSP. All data was pseudonymized by the Cancer Registry of Eastern Switzerland prior to analyses.

## Results

### Descriptives and comparative analyses

The study included 2558 women aged 50–69 with a BC diagnosis between 2010 and 2019. The average age at diagnosis was 59.3 years for women within and 60.7 years outside the MSP (see Table 1). Geographically, more BC cases are within the MSP in the canton of St. Gallen, compared to the more rural canton of Grisons (*p* < 0.05).

**Table 1:** Patient and tumor characteristics

	All observations		Within MSP		Outside MSP		Test for differences within versus outside MSP
	N	Mean (SD)/Proportion %	N	Mean (SD)/Proportion %	N	Mean (SD)/Proportion %	*p*-value
Age	2558	60.1 (6.0)	1057	59.3 (6.2)	1501	60.7 (5.9)	*p* < 0.05
Birthplace							*p* = 0.30
Outside Switzerland	669	26.2%	265	25.1%	404	26.9%	
In Switzerland	1889	73.8%	792	74.9%	1097	73.1%	
Canton							*p *< 0.05
Grisons	726	28.4%	258	24.4%	468	31.2%	
St. Gallen	1832	71.6%	799	75.6%	1033	68.8%	
Stage distribution							*p* < 0.05
In-Situ	316	12.4%	185	17.5%	131	8.7%	
I	987	38.6%	491	46.5%	496	33.0%	
II	821	32.1%	290	27.4%	531	35.4%	
III	231	9.0%	66	6.2%	165	11.0%	
IV	203	7.9%	25	2.4%	178	11.9%	
Mean tumor size (in mm)	2390	22.7 (18.9)	1012	19.3 (16.4)	1378	25.3 (20.3)	*p* < 0.05
Tumor Size < 2 cm	1307	54.7%	659	65.1%	648	47.0%	*p* < 0.05
Tumor Size < 5 cm	2208	92.4%	962	95.1%	1246	90.4%	*p* < 0.05
Lymph nodes involvement							*p* < 0.05
Positive (N1+)	907	35.7%	275	26.1%	632	42.4%	
Negative (N0)	1637	64.3%	778	73.9%	859	57.6%	
Hormone receptor							*p* = 0.21
Positive	2146	90.8%	872	91.7%	1274	90.2%	
Negative	218	9.2%	79	8.3%	139	9.8%	
Estrogen receptors							*p* = 0.19
Positive	2135	90.5%	868	91.5%	1267	89.9%	
Negative	224	9.5%	81	8.5%	143	10.1%	
Progesterone receptors							*p* < 0.05
Positive	1774	76.5%	740	78.6%	1034	75.0%	
Negative	546	23.5%	201	21.4%	345	25.0%	
Ki-67 proliferation index							*p* < 0.05
High (i.e. ≥ 25%)	825	38.7%	293	34.4%	532	41.5%	
Low (i.e. < 25%)	1307	61.3%	558	65.6%	749	58.5%	
Grading							*p* < 0.05
Grade I	367	16.7%	200	23.0%	167	12.6%	
Grade II	1170	53.3%	464	53.3%	706	53.2%	
Grade III	660	30.0%	206	23.7%	454	34.2%	
HER2 over expression							*p* = 0.58
Positive	341	15.5%	128	14.9%	213	15.8%	
Negative	1863	84.5%	729	85.1%	1134	84.2%	

Wilcoxon–Mann Whitney rank sum tests or student’s t-tests, as appropriate, were used to test for significance in differences

**Fig. 2:**
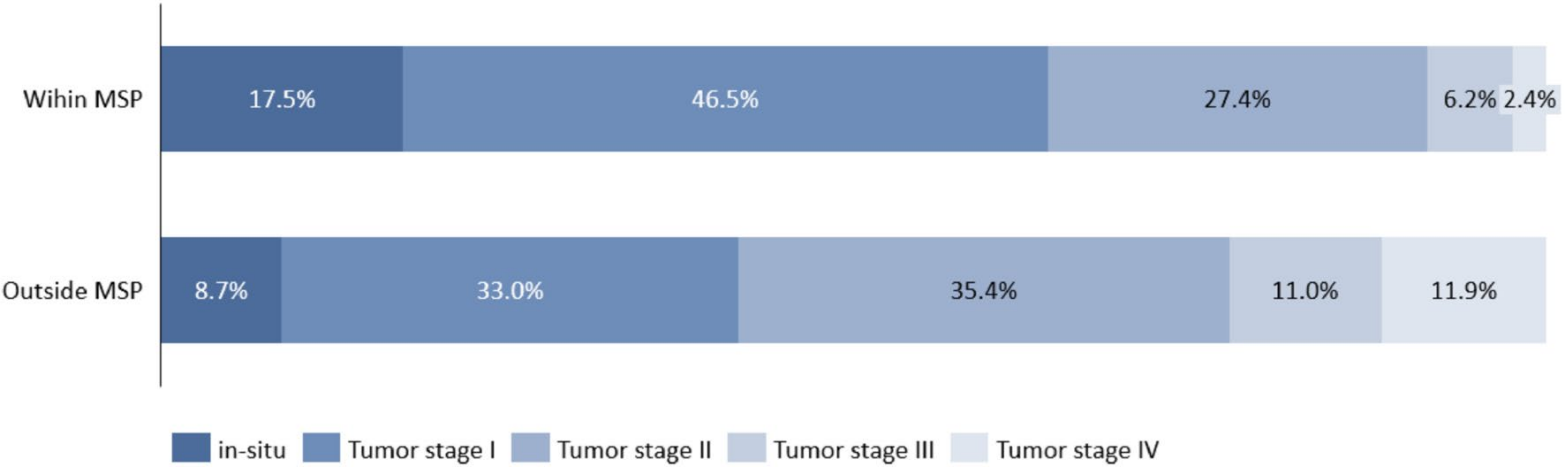
Tumor stage distribution of BC diagnoses within versus outside the MSP

While ~ 40% of BC cases in our sample were detected within the MSP, the share of detection varied considerably across the years, from 7% in the inauguration year of the program (2010) to 45–53% in the last two rounds from 2015 to 2019 (see Fig. 5). Tumor stage distribution of women within the MSP was significantly different from women outside the MSP (*p* < 0.05). Figure 2 displays the corresponding distributions and shows that BC within the MSP were diagnosed at earlier stages with fewer locally advanced and metastatic characteristics. In particular, within the MSP 17.5% of BC were in the in-situ stage (vs. 8.7% outside the MSP) and further 46.5% were detected in stage I (vs. 33.0%). Finally, only 6.2 and 2.4% of BC cases within the MSP had stages III and IV (vs. 11.0 and 11.9%). Moreover, lymph node involvement (26.1 vs. 42.4%) and tumor sizes (19.3 vs. 25.3 mm) were significantly different. Other significant differences between the two groups could be found for positivity of progesterone receptors (78.6% vs. 75.0%), Ki-67 (34.4% vs. 41.5%) and the distribution of the histologic grading—with a more favorable grading for BC within compared to outside the MSP.

### Comparative treatment and survival analyses

As can be seen in Table 2, treatments of women with invasive BC (stages I–IV) within the MSP were less aggressive and less burdensome than for women outside the MSP, which is reflected by half the rate of radical mastectomies (8.8% vs. 18.4%; *p* < 0.05) and a significantly lower proportion of patients undergoing adjuvant chemotherapy (33.7% vs. 44.1%; *p* < 0.05). Treatment was significantly more often performed within certified breast centers for women within the MSP. Noteworthy, the share of treated women in certified breast centers has changed over time. For instance, the first breast centers were certified in 2012 and the overall fraction of patients treated in breast centers has increased from 2% in 2012 to 74% in 2019.

**Table 2:** Comparative analyses of treatment and survival data

	All observations		Within MSP		Outside MSP		Test for differences within versus outside MSP
	N	Proportion in %	N	Proportion in %	N	Proportion in %	*p*-value
Certified Breast Centers	526	23.5%	274	31.4%	252	18.4%	*p* < 0.05
Radical Mastectomy	329	14.7%	77	8.8%	252	18.4%	*p* < 0.05
Adjuvant Chemotherapy	898	40.1%	294	33.7%	604	44.1%	*p* < 0.05
Recurrence or metastasis in the follow-up period	272	12.1%	58	6.7%	214	15.6%	*p* < 0.05
Overall survival							
5-year	1585	89.6%	642	96.7%	943	85.1%	*p* < 0.05
10-year	558	78.9%	158	91.4%	400	72.1%	*p* < 0.05
Stage I							
5-year	743	97.0%	370	98.7%	373	95.4%	*p* < 0.05
10-year	261	90.6%	94	96.3%	168	85.8%	*p* < 0.05
Stage II							
5-year	622	94.6%	221	98.6%	402	92.5%	*p* < 0.05
10-year	223	83.6%	55	90.7%	169	80.4%	Not significant
Stage III							
5-year	162	86.5%	44	91.3%	120	84.7%	Not significant
10-year	59	68.8%	9	76.6%	51	66.3%	Not significant
Stage IV							
5-year	60	36.3%	10	49.1%	51	34.5%	Not significant
10-year	17	18.1%	3	36.4%	15	16.2%	Not significant

Survival rates are derived from Kaplan–Meier estimates. For other variables Wilcoxon–Mann Whitney rank sum tests or student’s t-tests, as appropriate, were used to test for significance in differences. We consider results as significant when the p-value < 0.05

Furthermore, Table 2 shows that the survival rates were significantly higher for women within the MSP. The difference in the survival rates increased over time, where 1-year survival rates differed by 2.9 percentage points (pp) (99.7% vs. 96.8%), the 5-year survival rate already differed by 11.6pp (96.7% vs. 85.1%) and the 10-year survival rate by 19.3pp (91.4% vs. 72.1%). Similar differences were also observed for the subgroups of tumor stage I and II. Other stages have a fairly low number of cases but show similar, albeit insignificant patterns.

### Kaplan–Meier curves

**Fig. 3:**
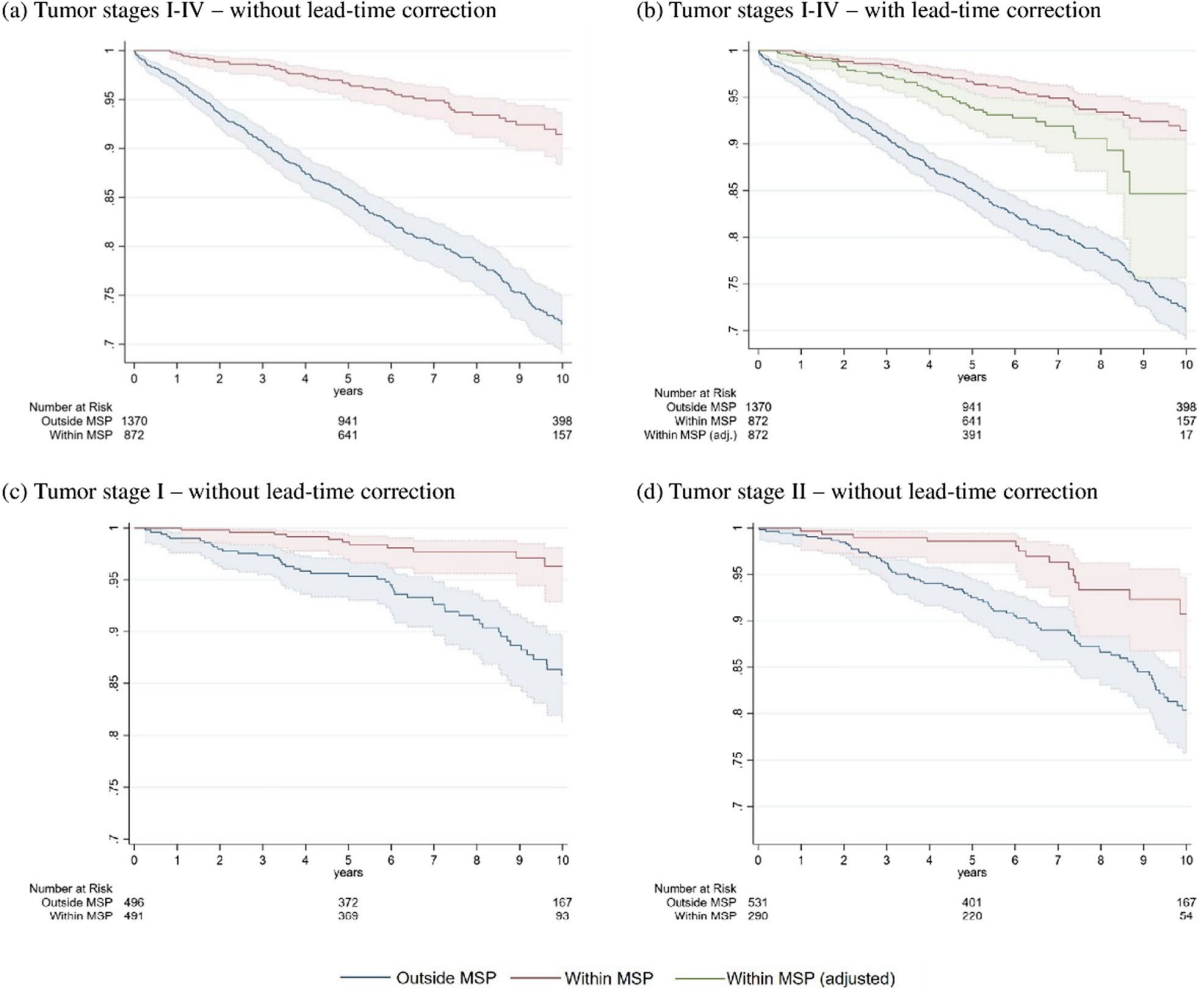
Kaplan–Meier Curves of overall survival of women within versus outside the MSP—for all tumor stages, lead-time corrected, stage I, and stage II. Note: Shaded areas represent the confidence interval; within MSP consists of screen-detected and interval carcinomas; outside MSP comprise all women with BC that were not participating in any MSP; within MSP (adjusted) corrects the survival time for lead time [13, 18]

Figure 3 depicts the survival probabilities over time. While the difference in survival increases over time, the differences are at almost all points in time significant. The overall Kaplan–Meier HR of within versus outside the MSP is 0.271 for all BC cases (Table 3). These significant better outcomes for women within the MSP persist, albeit at a lower magnitude, for the subgroups of stage I and II, that account for 71% of diagnosed BC cases. Moreover, Fig. 3b shows the lead-time corrected Kaplan–Meier curve for all tumor groups. The lead-time correction lowered the survival curve for women within the MSP, but survival was still significantly better for women within the MSP (84.7% survival rate after 10 years). Confidence intervals become large over time as sample size within MSP shrinks due to the lead-time correction.

**Table 3:** Hazard ratios for overall mortality calculated through cox proportional hazards regression models

Hazard ratios	Models					
	(1)	(2)	(3)	(4)	(5)	(6)
Screening program	0.271*** (0.197, 0.369)	0.377*** (0.277, 0.512)	0.394*** (0.290, 0.536)	0.550*** (0.389, 0.778)	0.691** (0.486, 0.983)	0.674** (0.471, 0.965)
Age			1.041*** (1.022, 1.060)	1.052*** (1.030, 1.074)	1.048*** (1.026, 1.071)	1.048*** (1.026, 1.072)
Ki-67 proliferation index				1.016*** (1.010, 1.024)	1.015*** (1.009, 1.021)	1.018*** (1.011, 1.024)
Tumor size				1.016*** (1.011, 1.021)	0.998 (0.991, 1.004)	0.996 (0.989, 1.021)
HER2						1.911*** (1.315, 2.777)
UICC TMN tumor stage (stage I)						
II					1.492** (1.058, 2.105)	1.495** (1.059, 2.110)
III					3.264*** (2.128, 5.007)	3.498*** (2.276, 5.376)
IV					15.500*** (10.502, 22.874)	16.965*** (11.407, 25.230)
Certified breast centers						1.391 (0.994, 1.936)
Control factors						
Lead-time corrected		x	x	x	x	x
Incidence-period corrected	x	x	x	x	x	x
Model statistics						
No. of observations	2242	2242	2242	1998	1998	1972
Proportional-hazards assumption holds	Yes	Yes	Yes	Yes	Yes	Yes
Prob > Chi2	0.000	0.000	0.000	0.000	0.000	0.000

Significance levels: ** *p* < 0.05, *** *p* < 0.01; Numbers in brackets represent the 95% confidence interval; ^a^Lead-time corrections are based on the methodological approach of Duffy et al. [13], using mean sojourn times from Tabar et al. [17]. ^b^Estimations are corrected for the year of incidence to account for treatment advancements over time

**Fig. 4:**
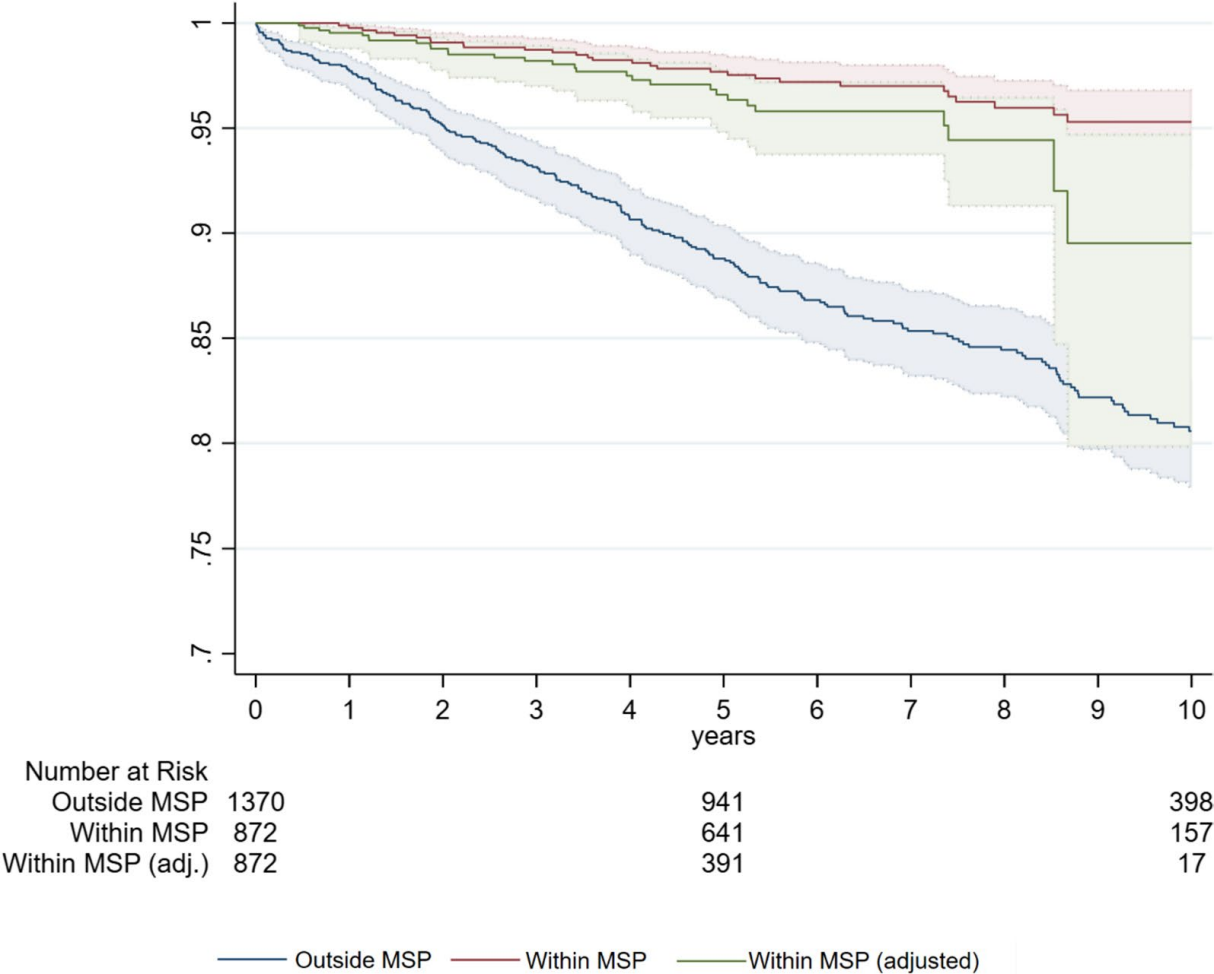
Kaplan–Meier Curves of BC related survival of women diagnosed within vs. outside the MSP. Notes: Shaded areas represent the confidence interval; within MSP consists of screen-detected and interval carcinomas; outside MSP comprise all women with BC that were not participating in any MSP; within MSP (adjusted) corrects the survival time for lead time [13, 18]

Furthermore, Fig. 4 shows, that the within MSP survival curve of BC specific survival is higher than for overall survival. For instance, 10-year BC survival lies at 96.5% for women within the MSP vs. at 92.5% for overall survival (82.5% vs. 74.0% for BC outside MSP). The Kaplan–Meier HR of BC related mortality of within MSP vs. outside MSP is 0.231 (see Table 3). This pattern is also true for the lead-time corrected Kaplan–Meier curve—which shows a survival rate at year 10 of 89.5%.

### Cox proportional hazards regressions

Table 3 displays HR for overall mortality from six specifications of the Cox proportional hazards regression model, from a simple specification (1&2) only including whether the woman participated in the MSP or not, to controlling for different variables (3–6). Specifications (2–6) show results for lead-time corrected survival times. Specification (1) shows that women within the MSP had a 72.9% lower hazard of mortality (HR: 0.271; *p* < 0.01). When only looking at BC-related mortality, the HR was 0.231 (*p* < 0.01) (see Table 4). The lead-time adjusted results (2) show a change of the HR to 0.377 (*p* < 0.01). The HR of the program remained similar when accounted for age (3). When controlled for tumor size and Ki-67 (4), control variables for length bias, the HR increases to 0.550 (*p* < 0.01) with significant negative effects of Ki-67 and tumor size on survival. The similar specification for BC-related survival showed an HR of 0.469 (*p* < 0.01).

**Table 4:** Hazard ratios for BC-related mortality calculated through cox proportional hazards regression models

Hazard ratios	Models					
	(1)	(2)	(3)	(4)	(5)	(6)
Screening program	0.231*** (0.154, 0.346)	0.313*** (0.212, 0.463)	0.318*** (0.215, 0.470)	0.469*** (0.294, 0.749)	0.688 (0.428, 1.108)	0.700 (0.433, 1.134)
Age			1.013 (0.991, 1.035)	1.016 (0.900, 1.044)	1.006 (0.978, 1.034)	1.007 (0.979, 1.035)
Ki-67 proliferation index				1.023*** (1.016, 1.031)	1.023*** (1.014, 1.031)	1.026*** (1.018, 1.035)
Tumor size				1.021*** (1.015, 1.026)	0.998 (0.991, 1.006)	0.996 (0.989, 1.003)
HER2						2.338*** (1.453, 3.763)
UICC TMN tumor stage (stage I)						
II					3.513*** (1.844, 6.690)	3.587*** (1.883, 6.836)
III					10.698*** (5.353, 21.379)	11.752*** (5.868, 23.537)
IV					60.610*** (31.589, 116.293)	69.953*** (36.149, 135.369)
Certified breast centers						1.440 (0.859, 2.415)
Control factors						
Lead-time corrected^a^		x	x	x	x	x
Incidence-period corrected^b^	x	x	x	x	x	x
Model statistics						
No. of observations	2242	2242	2242	1998	1998	1972
Proportional-hazards assumption holds	Yes	Yes	Yes	Yes	Yes	Yes
Prob > Chi2	0.000	0.000	0.000	0.000	0.000	0.000

Significance levels: ***p < 0.01; Numbers in brackets represent the 95% confidence interval; ^a^ Lead-time corrections are based on the methodological approach of Duffy et al. [13], using mean sojourn times from Tabar et al. [17]. ^b^Estimations are corrected for the year of incidence to account for treatment advancements over time

The effect of the MSP decreased when additionally accounted for tumor stages (HR: 0.691; *p* < 0.05). The higher the tumor stage, the higher was the hazard of mortality. Women diagnosed with stage IV had a ~ 16-times higher hazard of mortality than women with stage I carcinomas. When further controlled for HER2 and treatment in certified breast centers (6), the effect of the MSP changed slightly (HR: 0.699; *p* < 0.05), where HER2 had a significant effect on survival (HR: 1.880; *p* < 0.01), and certified breast center had no significant effect on the HR. HRs for BC-related mortality are shown in Table 4, and show insignificant HRs for the MSP as soon as tumor stages are included in the model.

## Discussion

The results demonstrate a statistically more favorable tumor stage distribution within compared to outside the MSP. Tumor stage is predictive for survival and women with lower stages had less aggressive treatment options available. We found, furthermore, that the proportion of Ki-67 levels is significantly higher for BC diagnosed outside the MSP. High Ki-67 levels are associated with higher grading, larger tumor size [19] and correlate to late endocrine resistance [20]. Consequently, we found that the number of mastectomies for cases within the MSP was about half compared to outside the MSP (8.9% vs. 17.7%). These results are in line with Hermann et al. [21] in a partially overlapping population covering a different time period. Less invasive and aggressive treatments directly translate into an improved quality of life for patients, as reported by Jay et al. [22] who showed better patient reported outcomes with respect to breast, psychosocial and sexual well-being of breast conserving therapy compared to mastectomy. Moreover, less lymph node infiltrations lead to fewer axillary dissections, potentially preventing possible burdensome side effects, such as lymphedema.

Most importantly, we found a significant survival difference for women within the MSP compared to women outside the MSP, starting in the first year after diagnosis with a continuously widening survival difference in absolute terms. At ten years after diagnosis the uncorrected survival difference amounted to 18.2pp (92.2% vs. 74.0%). However, estimations for survival effects of MSPs in observational studies are prone to lead-time bias [23]. Lead-time bias happens when screening increases the perceived survival time without affecting the course of the disease [24]. When correcting the survival of women with screen-detected BC for lead-time [13] the positive effect of MSPs on survival was still highly significant (HR: 0.377; *p* < 0.01). HR of the MSP, uncorrected and corrected for lead-time were in line with other studies [13, 25]. For instance, Schumann et al. [25] found an unadjusted HR of a German MSP of 0.23, and when adjusted for lead-time bias via several methods HRs between 0.32 and 0.39. Duffy et al. [13] estimated lead-time corrected HR for a Swedish MSP of 0.40. Nevertheless, other studies such as Munck et al. [14] reported lower effects of MSPs (HR: 0.76) in the Netherlands on disease-free survival when corrected for lead-time bias. This difference might be explained by a shorter follow-up time, investigating disease-free survival instead of overall survival, and by different patient characteristics in their sample (e.g., tumor stage distribution).

Furthermore, a different age distribution between the groups could affect overall survival, due to non-BC related mortality. Accounting for age only marginally affected survival. Further, differences in other characteristics such as Ki-67 and tumor size—potential determinants for a length bias (screening detects disproportionately more slowly growing tumors) [26]—could lead to an overestimation of the effectiveness of MSPs. However, lead-time corrected HR controlled for age, Ki-67 and tumor size still showed significantly positive effects of the MSP (HR: 0.550; *p* < 0.01).

This sustained difference in survival can have multiple reasons. Firstly, we observed different tumor characteristics of women depending on MSP participation. For instance, a more favorable tumor stage distribution is a well-recognized prognostic factor [27] and an acknowledged advantage of screening [28], and might explain the survival differences. While we showed that the detection of BC at an earlier stage had a significant effect on survival, it only partially explained the observed effects. Significant overall survival differences lowered but persisted when women of the same tumor stages were compared (e.g., for stage 1 at 10 years: 96.3% vs. 85.8%; *p* < 0.05), and when HR were controlled for tumor stage distribution (HR: 0.691; *p* < 0.05).

**Fig. 5:**
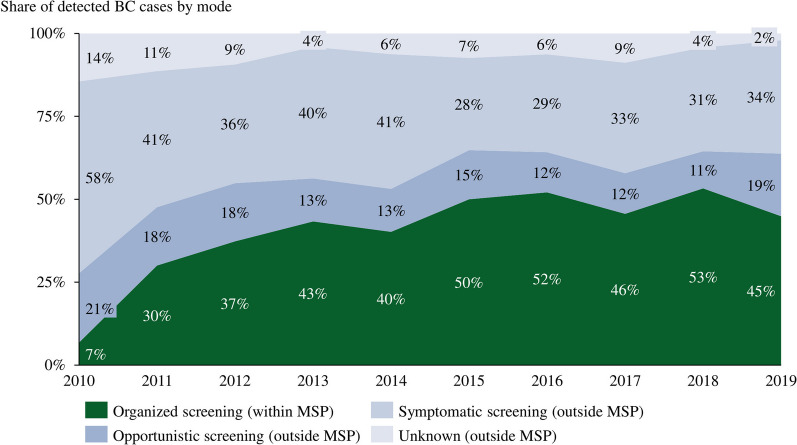
Share of detected BC cases within and outside MSP donna over time. Notes: Percentages are based on invasive carcinomas only and exclude in-situ carcinoma; Organized screening via the MSP “donna” was only started in 2010 in the cantons of St. Gallen and Grisons

Secondly, outcome differences might occur if groups are treated in different medical settings. We found that women within the MSP were more often treated in certified breast centers, where treatment was different with less mastectomies and fewer chemotherapy. These differences are supported by evidence from other studies. For example, a Dutch study found that non-screeners more often stopped taking adjuvant antihormonal treatments [29]. Moreover, if a suspicious finding appears in a screening mammography, the warranted sequence of further examinations, biopsy, and interdisciplinary tumor boards is still part of the organized MSP. Most of the time surgery and adjuvant treatment are given in one of the certified breast centers according to strict quality-controlled guidelines. The fact that women, whose BC was diagnosed outside the program, were more often treated outside of a certified breast center, could contribute to the different treatment outcomes and is a possible explanation for the finding that survival differences persisted in favor of the MSP even if corrected for stage distribution. Surprisingly, we did not find significant effects from treatments in certified breast centers for our sample. This result needs to be taken with care as certification only started in 2010 in Switzerland and their share varied considerably from 2010 to 2019. Furthermore, lower shares of detected BC cases within the program in earlier years (see Fig. 5) coupled with treatment advancements over time potentially bias our results. Therefore, we included an incidence year dummy in our regressions to account for period effects.

Lastly, as this was an observational study, there might have been unobserved differences in the characteristics of women participating in MSPs compared to those not participating. For instance, the screened population might differ in socioeconomic status (SES) and simultaneously in health-related factors as shown in several studies [30, 31], which can affect survival. A study from the Netherlands [30] showed that women of low SES were less likely to participate in MSPs than women with high SES (odds ratio of 1.8), and further had less favorable tumor stages—and thus were more likely to die from BC. Similar studies from Switzerland demonstrated significant differences of the tumor stage distribution of BC diagnosed in patients of lower compared to higher SES [32] as well as higher screening uptake of women with higher SES [33, 34]. While we controlled for tumor stage distribution in our analyses, other unobserved differences in survival relevant factors between the groups might have affected the differences in survival, and would, if systematic, lead to an overestimation of the survival effect from participating in MSPs.

Comparing our results to other studies shows that various incidence based mortality studies could find a 26% reduction of deaths of BC comparing to historical controls or areas without screening [11]. A more current survey of European screening programs showed a wide variation but significant reductions of BC mortality when comparing screened to non-screened women [12]. Duffy et al. [35] found that in the population of women regularly undergoing mammography screening the proportion of BC fatal after 10 years was reduced by 50%, somewhat less so in intermittent participants (− 36%). For women participating at five consecutive screening rounds the risk of dying from BC was reduced by 74%, and after statistical adjustments for self-selection to 66% (HR: 0.34) [36]. These HR are in the range of our findings.

### Limitations

Our study comes with limitations. Firstly, the overall mortality rate of age-standardized BC has significantly shrunk over the last 30 years not only internationally, but also in our local data from 34.3 to 18.7 per 100,000/year, whereas the age-standardized incidence has increased from 87.0 to 103.5 cases per 100,000/year [37]. Improvements in treatment had an important and beneficial effect on BC mortality and might be more relevant for decreasing overall mortality than screening [38]. In our study we do not compare with historical controls before the introduction of screening. Using incidence-based mortality data of the two groups diagnosed and treated at the same time in the same area eliminates a bias caused by considerably better treatment regimens over time. We controlled for period effects by including the year of the incidence as dummy variable to account for improvements in treatment over time. Secondly, the correction of lead-time bias can only approximate the effect of sojourn time and is based on a multi-state Markov model [13]. Abrahamsson et al. [39] in their simulation based on a tumor growth model showed that lead times can be shorter when conditional to tumor characteristics. This could potentially result in an underestimation of lead-time corrected survival effects from MSPs. Thirdly, adjustment of length bias is usually based on many assumptions [39]. By estimating models controlling for Ki-67 and tumor size, potential indicators for length bias, we showed that survival differences persisted. At the same time, differences in tumor size and Ki-67 are also direct advantages of MSPs—and controlled results do not fully reflect length bias. Lastly, bias might arise due to an overdiagnosis of tumors within the MSP [40]. By estimating our models excluding in-situ carcinoma we partially address the overdiagnosis bias [41]. Lastly, a certain share of BC cases outside the MSP are detected via opportunistic screening (see Fig. 5), which might affect the survival likelihood of the outside MSP cases positively. As we want to evaluate the effectiveness of an organized MSP, the “donna” program, compared to any outside detected BC, the key takeaways from our study are unaffected.

## Conclusion

To summarize, our results strongly support findings from other countries that participation in MSPs significantly increases survival of women with BC. Not correcting HR for lead-time and length bias leads to an overestimation of the effects from MSPs on survival. Moreover, diagnosis of BC in earlier tumor stages within MSPs allows for significantly less aggressive treatments contributing to a better quality of life. Given the presented results on the effectiveness of the MSP “donna” an evaluation of the cost-effectiveness is desirable. This might lead to a broader adoption of MSPs in Switzerland.

## Data Availability

Raw data for the mammography screening program “donna” and the Cancer Registry of Eastern Switzerland are not publicly available to preserve individuals’/patients´ privacy.
